# Divergent complication patterns of type 2 diabetes in African individuals who are lean versus overweight or obese: a multi-cohort analysis

**DOI:** 10.1007/s00125-026-06830-2

**Published:** 2026-08-23

**Authors:** Sabrina Esmail, Charles Hayfron-Benjamin, Samuel N. Darko, Ellis Owusu-Dabo, Erik Beune, Silver Bahendeka, Ayo P. Doumatey, Guanjie Chen, Charles Agyemang, Adebowale A. Adeyemo, Charles N. Rotimi, Karlijn A. C. Meeks, Felix P. Chilunga

**Affiliations:** 1 Department of Public & Occupational Health, Amsterdam Public Health Institute, Amsterdam UMC, Location AMC, University of Amsterdam, Amsterdam, the Netherlands; 2 Department of Physiology, University of Ghana Medical School, University of Ghana, Accra, Ghana; 3 Department of Molecular Medicine, Kwame Nkrumah University of Science and Technology, Kumasi, Ghana; 4 Department of Global and International Health, Kwame Nkrumah University of Science and Technology, Kumasi, Ghana; 5 Department of Medicine, Uganda Martyrs University School of Medicine, Kampala, Uganda; 6 Center for Research on Genomics and Global Health, National Human Genome Research Institute, National Institutes of Health, Bethesda, MD USA; 7 Division of Endocrinology, Diabetes and Metabolism, Department of Medicine, Johns Hopkins University School of Medicine, Baltimore, MD USA; 8 Division of Endocrinology, Diabetes and Nutrition, Department of Medicine, University of Maryland School of Medicine, Baltimore, MD USA

## Abstract

**Aims/hypothesis:**

Nearly 40% of African adults with type 2 diabetes are lean (BMI <25 kg/m^2^). Emerging evidence suggests that type 2 diabetes in African individuals who are lean may represent a distinct phenotype driven by impaired insulin secretion rather than insulin resistance, raising concerns about treatment mismatch and divergent disease outcomes. We examined complication profiles in Africans with type 2 diabetes who are lean vs overweight/obese.

**Methods:**

We analysed harmonised, individual-level cross-sectional data from two large, well-characterised African cohorts (Africa America Diabetes Mellitus study, *n*=2790; Research on Obesity and Diabetes among African Migrants study, *n*=541; total *n*=3331) of adults with type 2 diabetes from Ghana, Nigeria and Kenya. Participants were classified as lean (BMI <25 kg/m^2^) or overweight/obese (BMI ≥25 kg/m^2^). Robust Poisson regression, adjusted for age, sex, education and treatment, assessed associations with retinopathy, chronic kidney disease, stroke, hypertension and 10-year cardiovascular disease risk. Cohort-specific estimates were pooled using random effects, followed by mediation analysis examining contributions of lifestyle and metabolic markers to observed differences.

**Results:**

Type 2 diabetes in Africans who are lean was characterised by lower beta cell function and low insulin levels. Compared with individuals who are overweight/obese, adults who are lean showed a higher prevalence of retinopathy (pooled prevalence ratio [pPR] 1.36 [95% CI 1.13, 1.63]) and stroke (pPR 1.41 [95% CI 1.01, 1.99]), but lower hypertension (pPR 0.77 [95% CI 0.71, 0.85]) and 10-year cardiovascular disease risk (pPR 0.85 [95% CI 0.74, 0.97]). Chronic kidney disease prevalence did not differ between groups. Body fat percentage accounted for most differences (up to 92%).

**Conclusions/interpretation:**

Complication patterns in Africans with type 2 diabetes who are lean vs overweight/obese follow divergent paths. In Africa, where nearly 24 million people have type 2 diabetes, two-fifths of whom are lean, our findings add to growing evidence that lean type 2 diabetes may be a distinct phenotype, underscoring the urgent need for investigation into better-targeted management for this large, potentially mistreated, population.

**Graphical Abstract:**

**Figure:**
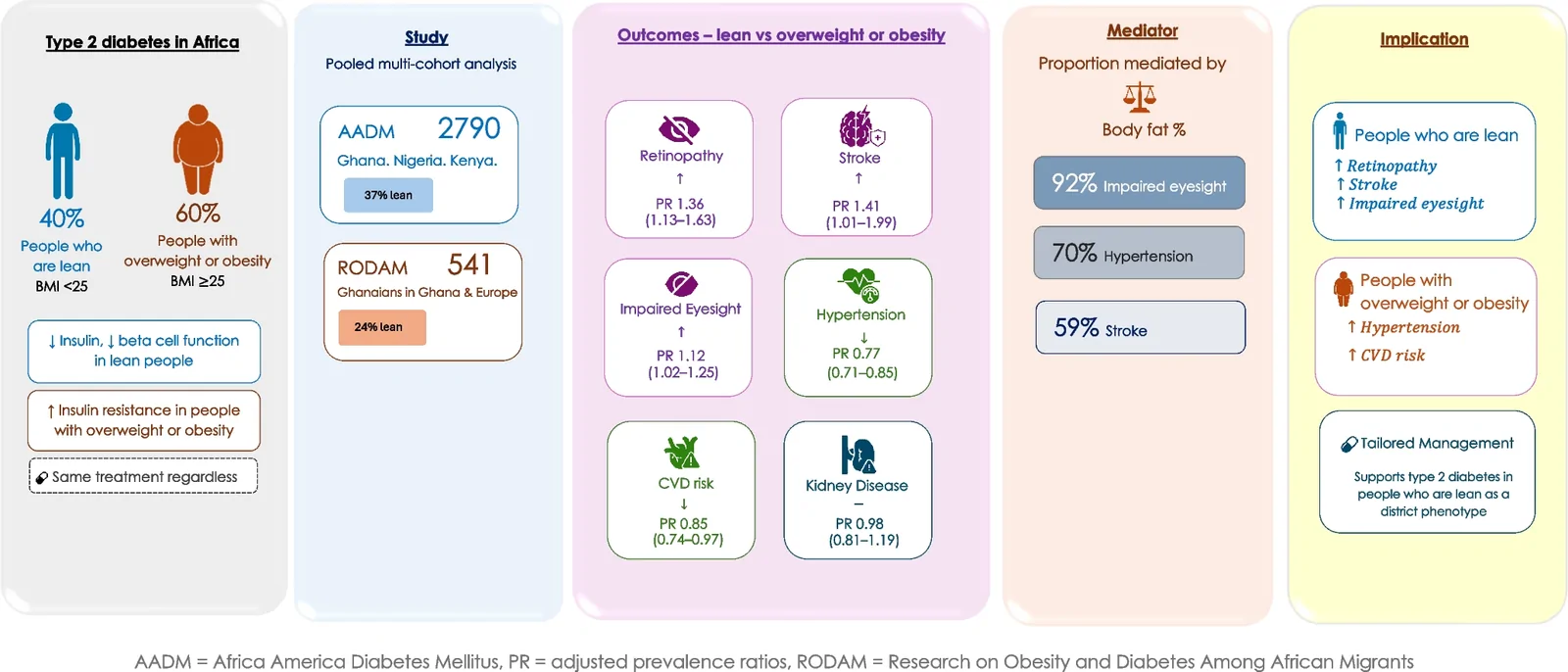

**Supplementary Information:**

The online version of this article (https://doi.org/10.1007/s00125-026-06830-2) contains peer-reviewed but unedited supplementary material.

**Figure:**
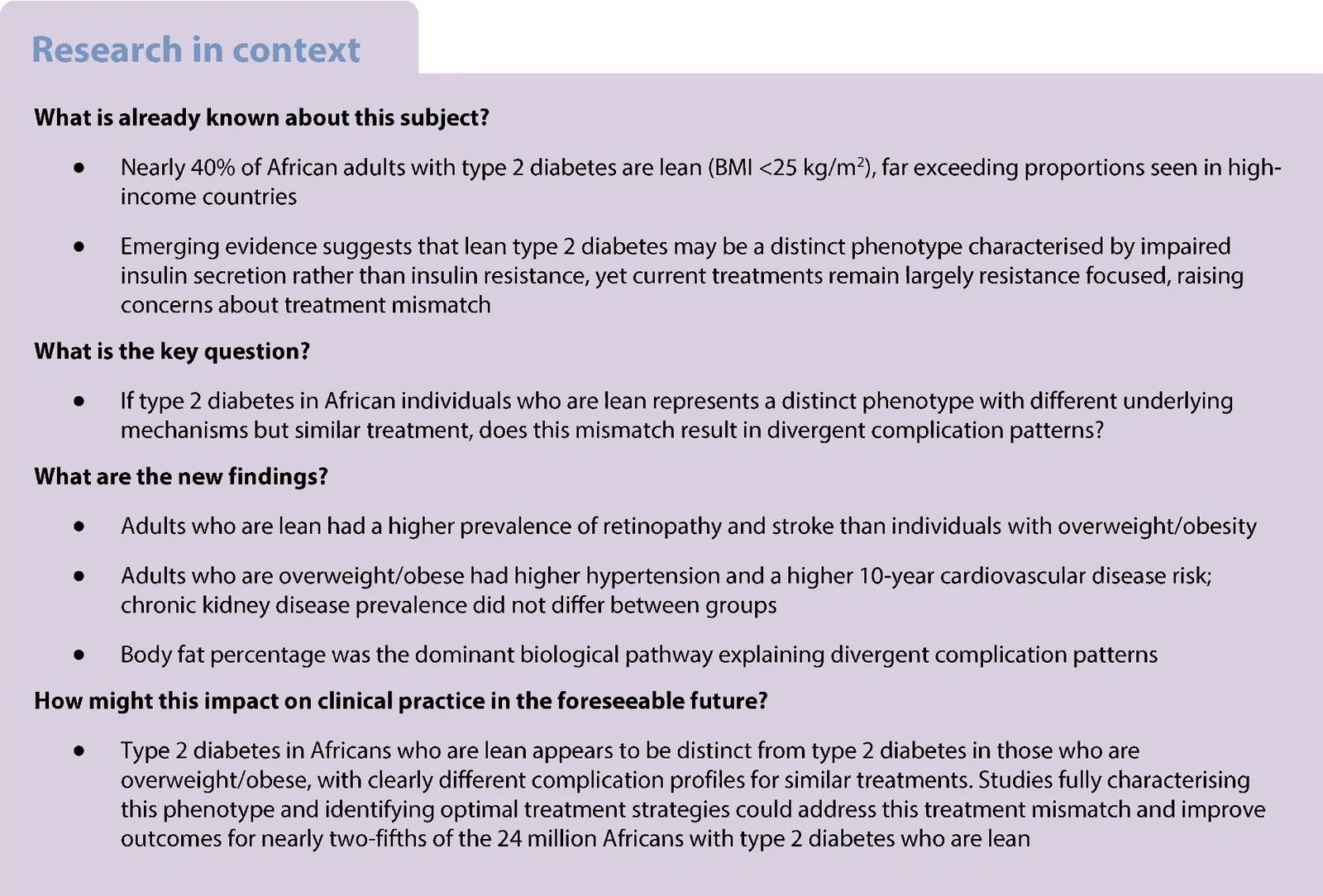

## Introduction

Over the past two decades, the prevalence of type 2 diabetes in sub-Saharan Africa has nearly doubled, becoming a significant public health concern [1, 2]. Intriguingly, almost half of the individuals categorised as having type 2 diabetes in sub-Saharan Africa are lean (BMI <25 kg/m^2^) [3, 4]. For example, our research among Ghanaians revealed that 60% of people with type 2 diabetes in rural areas and 40% of those in urban areas were lean [3]. In urban Uganda, the proportion of people with type 2 diabetes who are lean was nearly 35% [4]. Consistent with these findings, a meta-analysis of type 2 diabetes in African adults who are lean reported a pooled prevalence of 39% [5], and a global analysis categorised only 49% of individuals with type 2 diabetes in Africa as being obese [1]. This contrasts sharply with high-income countries, where obesity is present in roughly 90% of individuals with type 2 diabetes [6, 7]. Together, these data suggest that type 2 diabetes in African populations encompasses a substantial proportion of atypical forms and distinct subtypes [8, 9].

Emerging evidence indicates that risk factors for type 2 diabetes in people who are lean may differ from those in people who are overweight/obese [4, 10]. Early-life adversities (e.g. malnutrition and low birthweight) play a more prominent role in individuals who are lean [4, 10]. In contrast, unhealthy lifestyles (e.g. poor diet and physical inactivity) are more prevalent among individuals who are overweight/obese [4, 10]. This disparity may help explain the larger proportion of people with type 2 diabetes who are lean in rural Ghana (60%) than in urban areas (40%) [3], reflecting differences in early-life exposures vs lifestyle factors.

Furthermore, our study in Ghana [3] and studies from Uganda [4] have demonstrated that type 2 diabetes in individuals who are lean was predominantly characterised by reduced pancreatic beta cell secretory function rather than insulin resistance [4, 10]. Altogether, these findings suggest that type 2 diabetes in African individuals who are lean may represent a distinct phenotype that is potentially characterised by impaired insulin secretion rather than insulin resistance.

Despite this potential difference in underlying pathology, current guidelines in most African countries [11], largely adapted from World Health Organization (WHO) recommendations [11], recommend the initiation of type 2 diabetes therapy with metformin or sulfonylureas regardless of BMI or underlying mechanism. In people who are lean with impaired insulin secretion, however, reliance on insulin-sensitising drugs may not address underlying insulin deficiency, potentially leaving glucose levels suboptimal. This treatment mismatch may be associated with chronically elevated blood glucose, which may in turn be associated with greater microvascular and macrovascular damage. However, whether complication patterns differ between similarly treated individuals with fundamentally different underlying pathology has never been examined.

Identifying whether complication patterns differ in similarly treated people with distinct underlying pathology could help to confirm type 2 diabetes in people who are lean as a truly distinct phenotype. This confirmation could pave the way for tailored treatment strategies for close to two-fifths of the 24 million adults in Africa with type 2 diabetes (2021) [1], improving outcomes.

To address this gap, we assessed complication patterns and comorbidity profiles in Africans with type 2 diabetes who are lean compared to those who are overweight/obese. We analysed harmonised individual-level participant data from two large, well-characterised African cohorts: the Africa America Diabetes Mellitus (AADM) study, comprising adults residing in Ghana, Kenya and Nigeria [12], and the Research on Obesity and Diabetes among African Migrants (RODAM) study, comprising Ghanaian adults living in Ghana and Europe [13, 14].

## Methods

### Study design

We analysed harmonised individual-level participant cross-sectional data from two large, well-characterised cohorts: the AADM and RODAM studies [12, 13]. Both cohorts collected information on type 2 diabetes, obesity, diabetes complications and potential modifiers. Full study details have been described previously [12, 14–16]. Brief descriptions of both cohorts are provided in the electronic supplementary material (ESM) Methods (Brief Cohort Descriptions).

The AADM study was initiated to investigate genetic and environmental risk factors for type 2 diabetes and its complications in sub-Saharan Africa [12, 15, 16]. Between 1997 and 2016, participants with type 2 diabetes were recruited from outpatient medical clinics in major urban centres in Nigeria (Enugu, Ibadan and Lagos), Ghana (Accra and Kumasi) and Kenya (Eldoret). Adults aged ≥18 years were eligible. The multi-country design provided a unique opportunity to capture heterogeneity in exposures, clinical presentation and complications of diabetes across diverse African settings [12, 15, 16].

The RODAM study was conducted between 2012 and 2015 to uncover the relative contributions of genetic, environmental and lifestyle factors to obesity and diabetes, as well as their complications, among individuals of Ghanaian origin [14]. Recruitment took place in rural Ghana, in urban Ghana and among Ghanaian migrant communities in Amsterdam, Berlin and London. In Ghana, participants were randomly selected from community census lists, while in Europe recruitment was facilitated through municipal population registers, churches and Ghanaian community organisations. By including both local and migrant Ghanaians, the RODAM study enabled comparisons of environmental influences on diabetes risk and outcomes [14].

With respect to the representativeness of the study samples, both cohorts are broadly representative of their target populations, although each reflects the setting in which it was recruited. In the AADM study, eligible participants were aged 18 years and over, with or without type 2 diabetes, and the study aimed to enrol approximately equal numbers of men and women (the overall proportion of women was around 60%); the major ethnolinguistic groups represented (Yoruba, Ibo, Akan, Ga-Adangbe, Kalenjin, Kikuyu and Luhya) reflected those at the study sites [15, 16]. AADM participants were recruited from medical centres in urban settings and surrounding communities, so the sample is representative of patients receiving care in urban communities, who may differ in socioeconomic status, cardiometabolic factors and ethnicity from those in rural communities [15, 16]. In the RODAM study, random sampling from population registers facilitated representativeness; however, individuals not captured in these registers, such as undocumented migrants, may differ in their demographic and socioeconomic characteristics, and overall the RODAM sample is broadly representative of the target populations in terms of age and sex distribution [14].

### Ethical approval

Ethical approval was obtained from all participating countries in both cohorts [14, 15]. All participants provided written informed consent. Ethical clearance details have been published elsewhere [14, 15].

### Measurements

**Fig. 1:**
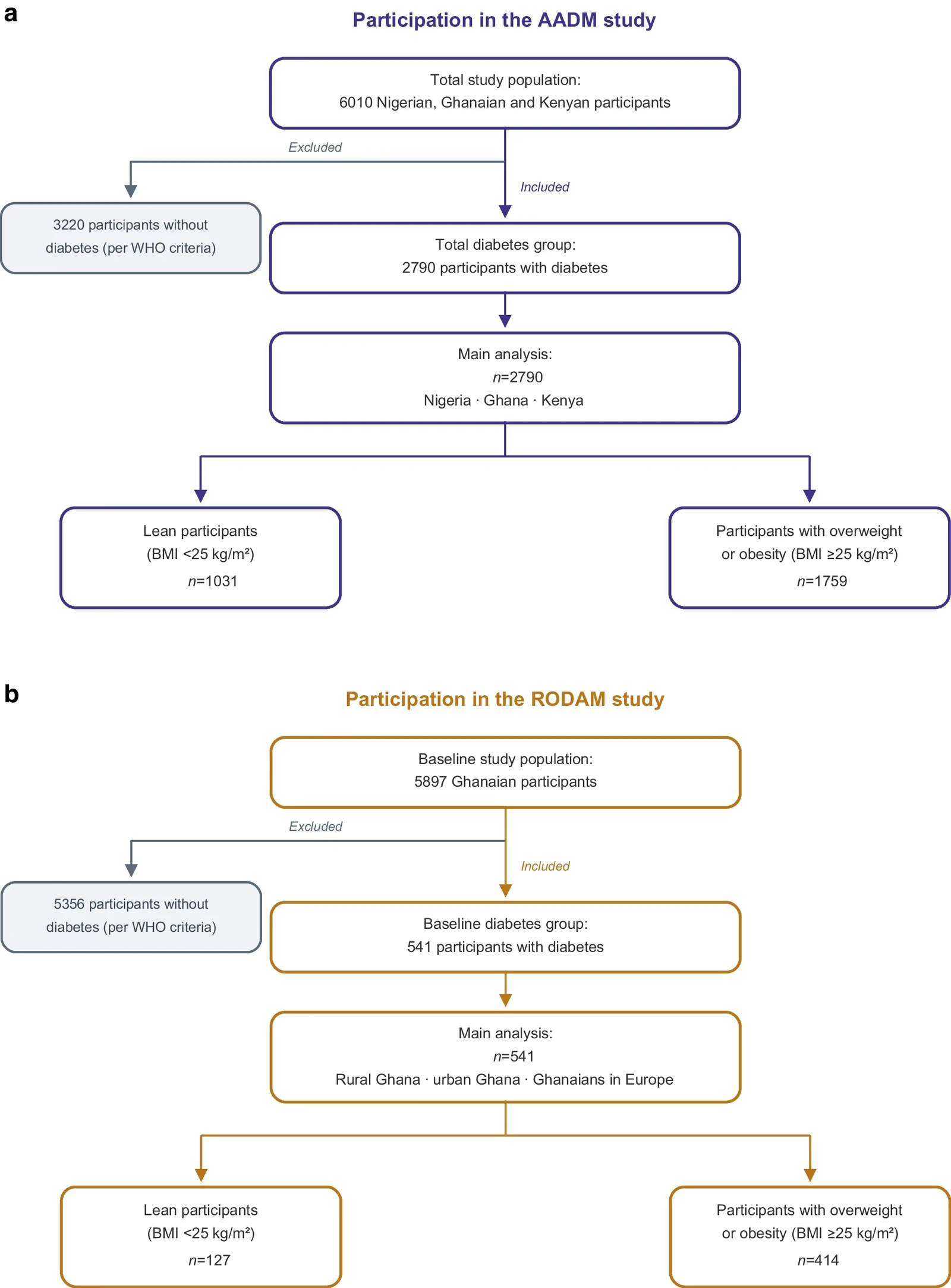
Flow chart of participation in the (**a**) AADM and (**b**) RODAM studies. Of 6010 AADM participants, 2790 had diabetes, of whom 1031 (37.0%) were lean and 1759 (63.0%) were overweight or obese. Of 5897 RODAM participants, 541 had diabetes, of whom 127 (23.5%) were lean and 414 (76.5%) were overweight or obese. Lean was defined as having a BMI of <25 kg/m^2^; overweight or obese was defined as having a BMI of ≥25 kg/m^2^

For this harmonised analysis, we restricted outcomes to those present in both cohorts, for replicability. Only adults with type 2 diabetes were included. In both the AADM and the RODAM studies, type 2 diabetes was defined as a fasting plasma glucose level of ≥7.0 mmol/l, having a previous physician diagnosis or using glucose-lowering medication. These criteria were met by 2790 participants in the AADM study and 541 participants in the RODAM study (Fig. 1). BMI was calculated from height and weight measured using stadiometers and calibrated scales in light clothing without shoes. Being lean was defined as having a BMI of <25 kg/m^2^, consistent with previous studies for comparability [3, 4, 10]. Of the AADM participants with type 2 diabetes, 1031 (37%) were lean (Fig. 1). Among the RODAM participants with type 2 diabetes, 127 (23.5%) were lean (Fig. 1). Body fat percentage (potential mediator) was measured using bioelectrical impedance analysis in both studies. Outcomes included stroke, chronic kidney disease (CKD), diabetic retinopathy, impaired eyesight, hypertension and 10-year cardiovascular disease (CVD) risk obtained via medical history or clinical measurement. Other measurements included age, sex (biological, self-reported), educational attainment, smoking status, alcohol use, BMI, waist-to-hip ratio, HOMA-IR, HOMA-B, lipids and type 2 diabetes treatment. A detailed description of the measurements and harmonisation is given in the ESM Methods (Measurements).

### Statistical analysis

Analyses were conducted in R version 4.3.2 (R Foundation for Statistical Computing, Vienna, Austria; https://www.r-project.org) (separately per cohort and then pooled). Participant characteristics were summarised by study group (lean vs overweight/obese). Continuous characteristics were expressed as medians (IQR) and categorical characteristics were expressed as counts (percentages). All variables had <15% missingness, except impaired eyesight in the AADM study (39%). Missing data were imputed using multiple imputation by chained equations (MICE; *mice package*). Multiple imputation yields less biased estimates than complete-case analysis [17].

Associations between BMI status and type 2 diabetes complications/comorbidities were estimated using Poisson regression with robust variance to obtain prevalence ratios (PRs). Three models were fitted: model 0 (unadjusted), model 1 (adjusted for age, sex and education) and model 2 (further adjusted for diabetes treatment). Cohort-specific estimates were then pooled using random effects (DerSimonian–Laird method; *meta package*), and between-study heterogeneity was quantified using the *I*^2^ statistic.

To explore pathways linking BMI status to complications, mediation analyses were performed (*mediation package*). Mediators included lifestyle (e.g. smoking), metabolic health (e.g. lipids), insulin levels and body composition (body fat percentage and waist-to-hip ratio). Body composition, particularly fat mass and waist-to-hip ratio, provides more precise insights into visceral fat distribution, clarifying the pathways to complications beyond general BMI [18].

In sensitivity analyses, we examined whether geographical site influenced results (site adjustments), as the RODAM cohort included participants from both Europe and Ghana. Additional models were not conducted in the AADM study, as participants were all from Africa.

## Results

### Participant characteristics

Overall, 3331 participants with type 2 diabetes were included (2790 from the AADM study and 541 from the RODAM study). Of these, 1158 (35%) were lean and 2173 (65%) were overweight or obese.

In the AADM study, 1031 (37.0%) of the participants with type 2 diabetes were lean and 1759 (63.0%) were overweight or obese. Most (56.5%) were recruited from Nigeria, 28.2% were recruited from Ghana and 15.3% were recruited from Kenya. The median age was 56 years. Men made up a greater proportion in the lean group than in the overweight/obesity group (53.9% vs 30.8%, respectively). When comparing the lean group with the overweight/obesity group, educational attainment did not differ between the groups. Tobacco smoking was more common among individuals who were lean, while alcohol consumption did not differ. Clinically, participants who were lean had lower median waist-to-hip ratios (0.90 vs 0.94), lower body fat percentages (22.5% vs 37.9%) and lower blood pressure (132.1/79.5 mmHg vs 139.2/83.5 mmHg). Cholesterol, lipid fractions and kidney function were comparable (Table 1).

**Table 1:** Characteristics of participants with type 2 diabetes in the AADM and RODAM studies

Variable	AADM study			RODAM study		
	Total (*N*=2790)	Lean (*N*=1031)	Overweight/obese (*N*=1759)	Total (*N*=541)	Lean (*N*=127)	Overweight/obese (*N*=414)
Demographics						
Age, years, median (IQR)	56 (48–64)	56 (48–64)	56 (48–63)	54 (48–60)	55 (47–61)	53 (48–59)
Sex, *n* (%)						
Male	1098 (39.4)	556 (53.9)	542 (30.8)	239 (44.2)	72 (56.7)	167 (40.3)
Female	1692 (60.6)	475 (46.1)	1217 (69.2)	302 (55.8)	55 (43.3)	247 (59.7)
Site, *n* (%)						
Ghana	786 (28.2)	259 (25.1)	527 (30.0)	191 (35.3)	82 (64.6)	109 (26.3)
Nigeria	1577 (56.5)	602 (58.4)	975 (55.4)	NA	NA	NA
Kenya	427 (15.3)	170 (16.5)	257 (14.6)	NA	NA	NA
European sites	NA	NA	NA	350 (64.7)	45 (35.4)	305 (73.7)
Education, *n* (%)						
Never been to school/primary	1451 (52.0)	547 (53.0)	904 (51.4)	175 (32.3)	48 (37.8)	127 (30.7)
Lower secondary	714 (25.6)	243 (23.6)	471 (26.8)	195 (36.1)	48 (37.8)	147 (35.5)
Upper secondary	193 (6.9)	76 (7.4)	117 (6.6)	110 (20.3)	20 (15.7)	90 (21.7)
Higher vocational/university	432 (15.5)	165 (16.0)	267 (15.2)	61 (11.3)	11 (8.7)	50 (12.1)
Lifestyle factors						
Smoking, *n* (%)						
Yes	79 (2.8)	38 (3.7)	41 (2.3)	32 (5.9)	6 (4.7)	26 (6.3)
No, never smoked	2289 (82.1)	786 (76.2)	1503 (85.5)	446 (82.5)	108 (85.1)	338 (81.6)
No, used to smoke	422 (15.1)	207 (20.1)	215 (12.2)	63 (11.6)	13 (10.2)	50 (12.1)
Alcohol consumption, *n* (%)						
Any	691 (24.8)	235 (22.8)	456 (25.9)	212 (39.2)	50 (39.4)	162 (39.1)
None/never	2099 (75.2)	796 (77.2)	1303 (74.1)	329 (60.8)	77 (60.6)	252 (60.9)
Clinical measurements, median (IQR)						
BMI, kg/m^2^	26.62 (23.38–30.28)	22.41 (20.58–23.84)	29.26 (27.00–32.64)	28.33 (25.13–31.84)	22.48 (20.73–23.93)	29.80 (27.38–32.83)
Systolic blood pressure, mmHg	136.5 (122.1–153.0)	132.1 (118.5–148.2)	139.2 (124.1–156.4)	136.5 (124.5–148.0)	134.5 (121.5–146.8)	138.0 (125.6–149.3)
Diastolic blood pressure, mmHg	82.3 (74.4–90.1)	79.5 (71.5–88.3)	83.5 (76.2–91.3)	84.3 (77.5–91.5)	82.5 (72.3–91.5)	84.5 (78.5–92.6)
Waist-to-hip ratio	0.92 (0.88–0.97)	0.90 (0.86–0.95)	0.94 (0.89–0.98)	0.95 (0.91–0.99)	0.92 (0.88–0.95)	0.96 (0.91–1.00)
Body fat percentage, %	32.72 (23.70–40.64)	22.50 (15.58–29.60)	37.90 (30.83–43.81)	32.12 (24.59–38.64)	20.85 (17.20–27.76)	35.60 (27.75–40.31)
Total cholesterol, mmol/l	5.11 (4.27–6.03)	4.97 (4.11–5.85)	5.20 (4.37–6.10)	4.99 (4.24–5.92)	5.05 (4.23–5.89)	4.98 (4.24–5.93)
Triglycerides, mmol/l	1.13 (0.87–1.58)	1.03 (0.78–1.41)	1.19 (0.93–1.68)	1.09 (0.79–1.46)	1.05 (0.84–1.46)	1.11 (0.79–1.47)
HDL-cholesterol, mmol/l	1.09 (0.83–1.37)	1.14 (0.88–1.42)	1.06 (0.78–1.34)	1.25 (1.04–1.45)	1.25 (1.02–1.46)	1.25 (1.04–1.45)
LDL-cholesterol, mmol/l	3.31 (2.61–4.09)	3.19 (2.48–3.96)	3.39 (2.66–4.16)	3.17 (2.51–3.92)	3.22 (2.46–3.83)	3.14 (2.51–3.93)
CKD-EPI (2021)_eGFR, ml/min per 1.73 m^2^	90.72 (72.07–108.83)	90.39 (72.18–109.05)	90.78 (72.06–108.71)	90.94 (77.81–103.37)	92.23 (75.35–105.77)	90.41 (78.43–102.29)

Lean is defined as having a BMI of <25 kg/m^2^. Overweight/obese is defined as having a BMI of ≥25 kg/m^2^

NA, not applicable

In the RODAM study, 127 (23.5%) were lean and 414 (76.5%) were overweight or obese. Thirty-five per cent of participants were recruited in Ghana and 65% were recruited in Europe (Amsterdam, Berlin and London). The median age was 55 years in individuals who were lean and 53 years in those who were overweight/obese. Men made up a greater proportion in the lean group than in the overweight/obesity group (56.7% vs 40.3%, respectively). When comparing the lean group with the overweight/obesity group, higher education was more common in participants who were overweight/obese (12.1% vs 8.7%). Participants who were overweight/obese smoked more tobacco (6.3% vs 4.7%), while alcohol consumption did not differ. Clinically, participants who were lean had lower median waist-to-hip ratios (0.92 vs 0.96), lower body fat percentages (20.85% vs 35.60%) and lower blood pressure (134.5/82.5 mmHg vs 138.0/84.5 mmHg). Cholesterol, lipid fractions and kidney function were also comparable (Table 1).

### Glycaemic parameters and diabetes management

In the AADM study, glucose and type 2 diabetes measures differed between groups (Table 2). When comparing the lean group with the overweight/obesity group, median fasting glucose was higher in participants who were lean (9.4 vs 8.4 mmol/l). Among participants not receiving insulin therapy, median insulin was lower in the lean group (57 vs 74 pmol/l), as were HOMA-IR (4.0 vs 4.6) and HOMA-B (42 vs 60). Most participants had a prior type 2 diabetes diagnosis (99.5%) and were on treatment: oral tablets were more common among those who were overweight/obese (84.8% vs 79.2%), while insulin use was more frequent in participants who were lean (22.7% vs 15.9%). Hypertension was more prevalent in participants who were overweight/obese (76.2% vs 58.8%), while participants who were lean had a higher prevalence of stroke (3.0% vs 2.2%) and diabetic retinopathy (12.9% vs 10.1%). The 10-year CVD risk was higher in the overweight/obesity group (18.4% vs 15.6%), and CKD prevalence was similar across groups (Table 2).

**Table 2:** Type 2 diabetes management, control, comorbidities and complications in the AADM and RODAM studies

Variable	AADM study			RODAM study		
	Total (*N*=2790)	Lean (*N*=1031)	Overweight/obese (*N*=1759)	Total (*N*=541)	Lean (*N*=127)	Overweight/obese (*N*=414)
Glucose levels, median (IQR)						
Glucose, mmol/l	8.67 (6.11–12.50)	9.42 (5.94–13.94)	8.39 (6.17–11.72)	7.45 (5.85–10.67)	8.04 (5.97–12.17)	7.39 (5.79–9.97)
Insulin, pmol/l^a^	66.3 (38.4–120.1)	56.5 (29.3–105.3)	73.8 (43.8–132.0)	43.8 (26.4–66.6)	27.6 (16.5–45.1)	49.2 (31.8–71.9)
HOMA-IR^a^	4.40 (2.17–9.11)	4.04 (1.84–8.48)	4.62 (2.47–9.37)	2.61 (1.34–4.41)	1.62 (0.85–3.07)	2.75 (1.54–4.79)
HOMA-B^a^	53.57 (22.40–122.19)	41.52 (16.20–106.36)	59.72 (27.39–129.75)	38.92 (19.48–73.45)	21.92 (10.74–45.61)	43.08 (25.74–79.77)
Diagnosis and management of type 2 diabetes, *n* (%)						
Previously diagnosed with diabetes	2775 (99.5)	1026 (99.5)	1749 (99.4)	384 (71.0)	84 (66.1)	300 (72.5)
Treated for diabetes in the past	2710 (97.1)	1006 (97.6)	1704 (96.9)	354 (65.4)	66 (52.0)	288 (69.6)
Using tablets for diabetes	2309 (82.8)	817 (79.2)	1492 (84.8)	309 (57.1)	68 (53.5)	241 (58.2)
On a diabetic diet	2747 (98.5)	1009 (97.9)	1738 (98.8)	287 (53.0)	60 (47.2)	227 (54.8)
Using insulin injections	513 (18.4)	234 (22.7)	279 (15.9)	155 (28.7)	28 (22.0)	127 (30.7)
Comorbidities and complications						
10-year CVD risk, %, median (IQR)	17.4 (9.5–25.3)	15.6 (8.6–25.3)	18.4 (10.1–25.3)	16.3 (9.4–21.2)	14.4 (9.8–23.8)	16.9 (9.3–20.4)
Hypertension, *n* (%)	1946 (69.7)	606 (58.8)	1340 (76.2)	374 (69.1)	76 (59.8)	298 (72.0)
Stroke, *n* (%)	70 (2.5)	31 (3.0)	39 (2.2)	47 (8.7)	18 (14.2)	29 (7.0)
Diabetic retinopathy, *n* (%)	311 (11.1)	133 (12.9)	178 (10.1)	230 (42.5)	69 (54.3)	161 (38.9)
Impaired eyesight, *n* (%)	1511 (54.2)	596 (57.8)	915 (52.0)	69 (12.8)	22 (17.3)	47 (11.4)
CKD, *n* (%)	426 (15.3)	150 (14.5)	276 (15.7)	51 (9.4)	9 (7.1)	42 (10.1)

Lean is defined as having a BMI of <25 kg/m^2^. Overweight/obese is defined as having a BMI of ≥25 kg/m^2^

^a^Insulin, HOMA-IR and HOMA-B were assessed only in participants not receiving insulin therapy (AADM study, *n*=2277 [lean, *n*=797; overweight/obese, *n*=1480]; RODAM study, *n*=386 [lean, *n*=99; overweight/obese, *n*=287])

In the RODAM study, similar patterns emerged (Table 2). When comparing the lean group with the overweight/obesity group, participants with type 2 diabetes who were lean had lower median insulin (28 vs 49 pmol/l), lower HOMA-IR (1.6 vs 2.8) and lower HOMA-B (22 vs 43). Fewer participants who were lean had a prior type 2 diabetes diagnosis (66.1% vs 72.5%) and fewer were on oral agents (53.5% vs 58.2%), while insulin therapy was more frequent in those who were overweight/obese (30.7% vs 22.0%). Hypertension was more prevalent in participants who were overweight/obese (72.0% vs 59.8%), while participants who were lean had a higher prevalence of stroke (14.2% vs 7.0%), diabetic retinopathy (54.3% vs 38.9%) and impaired eyesight (17.3% vs 11.4%). The 10-year CVD risk was higher in the overweight/obesity group (16.9% vs 14.4%), and CKD prevalence was lower in the lean group than the overweight/obesity group (7.1% in the lean group vs 10.1% in the overweight/obesity group; Table 2).

### Associations between BMI status and complications/comorbidities (study specific)

In the AADM study, participants with type 2 diabetes who were lean had a lower prevalence of hypertension than participants who were overweight/obese (adjusted PR [aPR] 0.77 [95% CI 0.70, 0.85]; Table 3). They also had a higher prevalence of diabetic retinopathy (aPR 1.39 [95% CI 1.10, 1.75]) and impaired eyesight (aPR 1.14 [95% CI 1.02, 1.27]). In those who were lean, the PR for stroke was elevated but the CI crossed the null (aPR 1.55 [95% CI 0.95, 2.54]), they had a lower prevalence of hypertension (aPR 0.77 [95% CI 0.70, 0.85]) and a lower 10-year CVD risk (aPR 0.83 [95% CI 0.70, 0.98]; Table 3), and no clear differences were observed for CKD (aPR 0.99 [95% CI 0.81, 1.21]).

**Table 3:** Associations between being lean and overweight/obese with type 2 diabetes and complications/comorbidities in the AADM and RODAM studies

Diabetes complications/comorbidities	AADM study			RODAM study		
	Model 0, PR (95% CI)	Model 1, PR (95% CI)	Model 2, PR (95% CI)	Model 0, PR (95% CI)	Model 1, PR (95% CI)	Model 2, PR (95% CI)
10-year CVD risk						
Overweight/obesity (ref)	Ref	Ref	Ref	Ref	Ref	Ref
Lean (normal weight)	0.90 (0.82–1.00)	0.86 (0.77–0.95)	0.83 (0.70–0.98)	0.94 (0.74–1.20)	0.88 (0.68–1.12)	0.88 (0.69–1.13)
Hypertension						
Overweight/obesity (ref)	Ref	Ref	Ref	Ref	Ref	Ref
Lean (normal weight)	0.77 (0.70–0.85)	0.77 (0.70–0.85)	0.77 (0.70–0.85)	0.83 (0.64–1.06)	0.81 (0.62–1.04)	0.81 (0.62–1.05)
Stroke						
Overweight/obesity (ref)	Ref	Ref	Ref	Ref	Ref	Ref
Lean (normal weight)	1.35 (0.84–2.17)	1.35 (0.82–2.19)	1.55 (0.95–2.54)	2.02 (1.16–3.52)	1.45 (0.90–2.27)	1.30 (0.81–2.09)
Eyesight						
Overweight/obesity (ref)	Ref	Ref	Ref	Ref	Ref	Ref
Lean (normal weight)	1.12 (1.01–1.24)	1.15 (1.03–1.27)	1.14 (1.02–1.27)	1.53 (0.96–2.43)	1.03 (0.78–1.36)	1.03 (0.77–1.36)
CKD						
Overweight/obesity (ref)	Ref	Ref	Ref	Ref	Ref	Ref
Lean (normal weight)	0.93 (0.77–1.11)	0.93 (0.75–1.13)	0.99 (0.81–1.21)	0.70 (0.35–1.40)	0.74 (0.33–1.51)	0.90 (0.42–1.94)
Diabetic retinopathy						
Overweight/obesity (ref)	Ref	Ref	Ref	Ref	Ref	Ref
Lean (normal weight)	1.36 (1.09–1.69)	1.30 (1.04–1.64)	1.39 (1.10–1.75)	1.40 (1.05–1.84)	1.46 (1.09–1.94)	1.31 (0.98–1.76)

Reference group: overweight/obese (BMI ≥25 kg/m^2^)

Poisson regression models with robust standard errors were used to estimate PRs and 95% CIs

Model 0 is unadjusted; model 1 is adjusted for age, sex and education; model 2 is additionally adjusted for diabetes treatment

Site in the RODAM study (Ghana vs Europe) was not included, as it reflects early-life and contextual exposures that are part of the causal pathway to lean vs obese diabetes; however, treatment was included to account for any site-related differences in care

Ref, reference group

In the RODAM study, patterns were directionally similar but less precise (Table 3). Participants who were lean did not differ meaningfully from those who were overweight/obese in hypertension (aPR 0.81 [95% CI 0.62, 1.05]) or 10-year CVD risk (aPR 0.88 [95% CI 0.69, 1.13]). Retinopathy prevalence was higher in those who were lean, although the CI crossed the null (aPR 1.31 [95% CI 0.98, 1.76]), and no clear differences were seen for stroke (aPR 1.30 [95% CI 0.81, 2.09]), impaired eyesight (aPR 1.03 [95% CI 0.77, 1.36]) or CKD (aPR 0.90 [95% CI 0.42, 1.94]).

### Pooled associations between BMI status and complications/comorbidities

**Fig. 2:**
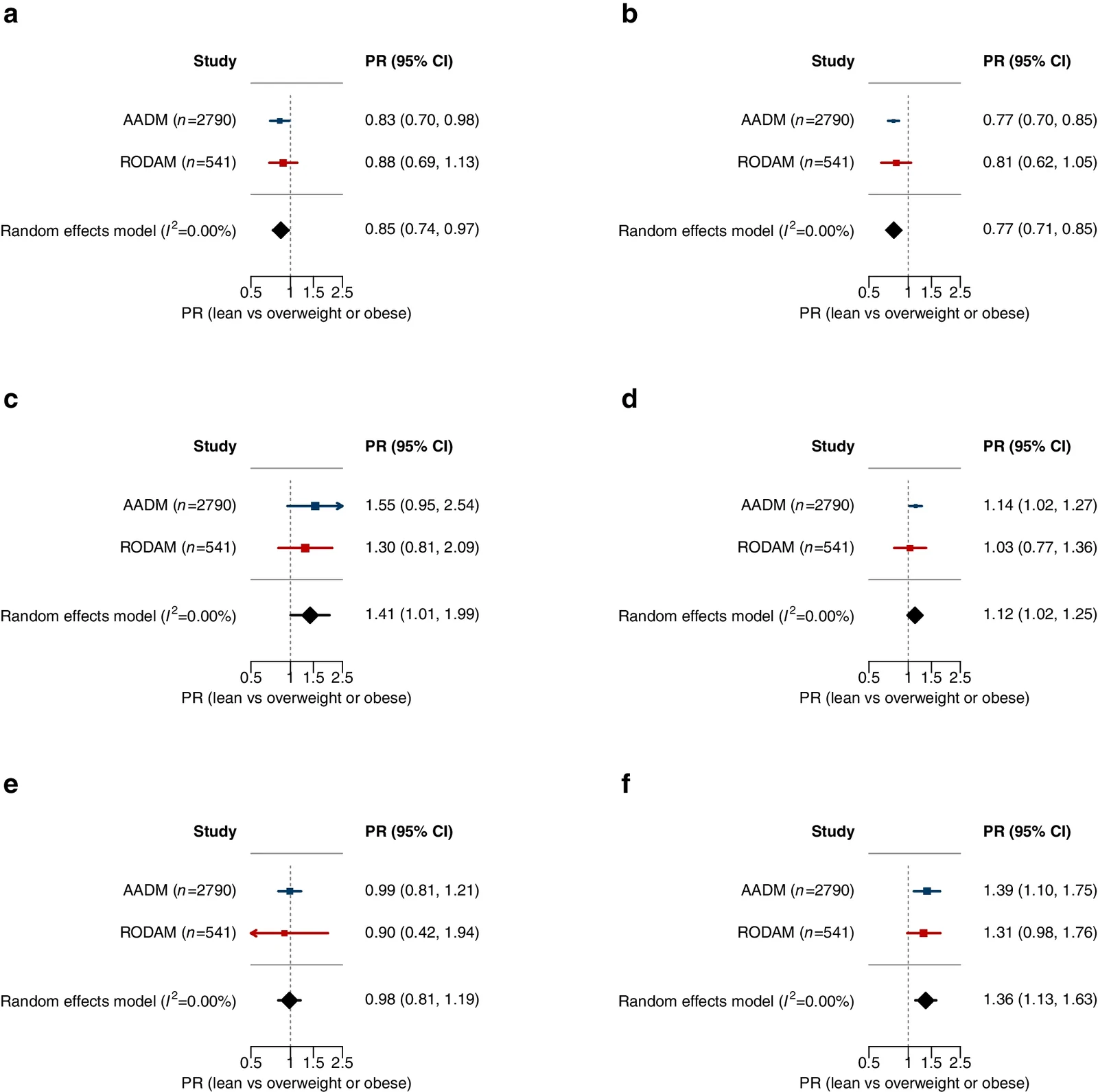
Pooled associations between BMI status and complications in Africans with type 2 diabetes. Individuals who were lean showed a higher prevalence of diabetic retinopathy, impaired eyesight and stroke than individuals who were overweight/obese, while the latter showed higher hypertension and 10-year CVD risk than the former; CKD did not differ between groups. PRs with 95% CIs are shown, comparing individuals who are lean (BMI <25 kg/m^2^) with those who are overweight or obese (BMI ≥25 kg/m^2^), adjusted for age, sex, education and diabetes treatment, for the following complications: (**a**) 10-year CVD risk; (**b**) hypertension; (**c**) stroke; (**d**) impaired eyesight; (**e**) CKD; and (**f**) diabetic retinopathy. Dashed vertical lines represent the null value (PR=1). Coloured squares represent cohort-specific estimates; diamonds represent the pooled random effects estimate. *I*^2^ is the heterogeneity statistic

In pooled analyses, distinct complication patterns emerged when comparing participants who were lean with those who were overweight/obese (Fig. 2). Individuals who were lean had a significantly higher prevalence of stroke (pooled PR [pPR] 1.41 [95% CI 1.01, 1.99], Fig. 2c) and diabetic retinopathy (pPR 1.36 [95% CI 1.13, 1.63], Fig. 2f) than those who were overweight/obese, consistent across both studies. Impaired eyesight was also more common among participants who were lean (pPR 1.12 [95% CI 1.02, 1.25], Fig. 2d). By contrast, hypertension was significantly less prevalent in individuals who were lean than in their overweight/obese counterparts (pPR 0.77 [95% CI 0.71, 0.85], Fig. 2b), while 10-year CVD risk was also lower in individuals who were lean (pPR 0.85 [95% CI 0.74, 0.97], Fig. 2a). No significant differences were observed for CKD (pPR 0.98 [95% CI 0.81, 1.19], Fig. 2e). Heterogeneity was close to zero in pooled analyses.

### Mediation analysis of lifestyle, anthropometry and lipids in BMI–complication associations

In the AADM study, body fat percentage was the dominant mediator, explaining up to 92% of differences in impaired eyesight and 59% of differences in stroke between participants who were lean and those who were overweight/obese (Table 4). Triglycerides and HDL-cholesterol mediated up to 78% and 48% of CKD differences, respectively. Although current smoking was more common in participants who were lean than in those who were overweight/obese (3.7% vs 2.3%, respectively), it explained less than 1% of the associations with diabetic retinopathy, impaired eyesight and stroke, and direct effects were essentially equal to total effects across these outcomes. All other lifestyle factors contributed minimally (<5%).

**Table 4:** Mediation of lifestyle factors, anthropometric measurements and insulin in the association between BMI status and diabetes-related complications/comorbidities in the AADM and RODAM studies

Mediator	AADM study				RODAM study			
	Indirect effect estimate	Direct effect estimate	Total effect estimate	Proportion mediated estimate, %	Indirect effect estimate	Direct effect estimate	Total effect estimate	Proportion mediated estimate, %
Outcome: 10-year CVD risk								
Lifestyle factors								
Never smoked	−0.000	−0.163*	−0.170*	0.3	−0.004	−0.135*	−0.143*	2.4
Used to smoke	0.000	−0.154*	−0.167*	0.0	−0.002	−0.128*	−0.136*	0.5
Clinical measurements								
Waist-to-hip ratio	−0.015*	−0.096*	−0.114*	13.0	−0.019	−0.088*	−0.110*	16.6
Body fat percentage	−0.009	−0.104*	−0.115*	7.6	−0.024	−0.081	−0.106*	22.2
Cholesterol	−0.001	−0.112*	−0.113*	0.8	0.002	−0.109*	−0.106*	1.4
LDL-cholesterol	−0.002	−0.110*	−0.113*	2.0	0.001	−0.100*	−0.100*	0.1
HDL-cholesterol	−0.012*	−0.099*	−0.114*	10.7	−0.001	−0.110*	−0.111*	1.2
Triglycerides	−0.024*	−0.091*	−0.119*	20.0	0.005	−0.113*	−0.107*	3.3
Insulin	0.000	−0.115*	−0.114*	0.3	−0.016	−0.087*	−0.106*	14.5
Outcome: CKD								
Lifestyle factors								
Never smoked	−0.000	−0.009	−0.009	0.2	0.001	−0.003	−0.002	0.6
Used to smoke	−0.000	−0.009	−0.009	0.0	0.000	−0.003	−0.003	0.2
Clinical measurements								
Waist-to-hip ratio	−0.004	−0.006	−0.009	15.6	0.008	−0.011	−0.004	1.3
Body fat percentage	0.009	−0.017	−0.009	37.5	0.014	−0.011	−0.002	0.4
Cholesterol	−0.003	−0.008	−0.011	13.5	0.000	−0.004	−0.004	0.1
LDL-cholesterol	−0.003	−0.008	−0.011	15.0	0.000	−0.004	−0.004	0.1
HDL-cholesterol	0.009*	−0.019	−0.010	47.6	−0.003	0.000	−0.003	4.2
Triglycerides	−0.015*	0.007	−0.008	77.8	0.001	−0.005	−0.004	−0.2
Insulin	0.001	−0.010	−0.010	3.1	−0.009	0.005	−0.005	20.6
Outcome: diabetic retinopathy								
Lifestyle factors								
Never smoked	0.000	0.023	0.023	0.0	−0.008	0.129*	0.121*	6.0
Used to smoke	0.000	0.022	0.022	0.0	−0.002	0.123*	0.121*	1.1
Clinical measurements								
Waist-to-hip ratio	−0.006*	0.030*	0.023	23.0	0.003	0.117*	0.119*	2.0
Body fat percentage	0.028*	−0.004	0.023	114.8^a^	0.014	0.106	0.120*	11.1
Cholesterol	−0.001	0.023	0.022	2.2	0.000	0.120*	0.120*	0.1
LDL-cholesterol	−0.001	0.023	0.022	3.4	0.000	0.120*	0.121*	0.0
HDL-cholesterol	0.008*	0.013	0.022	34.5	−0.003	0.121*	0.118*	1.9
Triglycerides	−0.003*	0.027*	0.023	12.1	0.004	0.117*	0.121*	2.8
Insulin	0.000	0.022	0.022	0.3	−0.022*	0.141*	0.118*	17.6
Outcome: eyesight								
Lifestyle factors								
Never smoked	0.000	0.071	0.071	0.0	−0.006	0.029	0.023	4.7
Used to smoke	0.000	0.071	0.071	0.0	−0.004	0.025	0.021	1.5
Clinical measurements								
Waist-to-hip ratio	−0.004	0.074*	0.070*	5.3	−0.011	0.031	0.021	9.4
Body fat percentage	0.066*	0.005	0.071*	92.1	0.049	−0.028	0.021	53.1
Cholesterol	0.001	0.071*	0.071*	0.7	0.001	0.022	0.023	1.8
LDL-cholesterol	0.001	0.072*	0.072*	0.5	0.000	0.022	0.022	0.9
HDL-cholesterol	−0.009*	0.079*	0.070*	12.8	−0.002	0.023	0.021	0.5
Triglycerides	0.004	0.067*	0.071*	6.0	0.000	0.023	0.023	0.0
Insulin	−0.001	0.071*	0.071*	0.5	−0.009	0.031	0.022	6.5
Outcome: stroke								
Lifestyle factors								
Never smoked	0.000	0.008	0.008	0.6	−0.008	0.056	0.046	11.6
Used to smoke	0.000	0.009	0.009	0.0	0.002	0.041	0.044	2.7
Clinical measurements								
Waist-to-hip ratio	0.000	0.008	0.008	2.2	−0.001	0.045	0.044	1.9
Body fat percentage	0.007	0.001	0.008	59.1	0.015	0.034	0.046	16.2
Cholesterol	0.000	0.008	0.008	0.4	0.000	0.043	0.044	0.1
LDL-cholesterol	0.000	0.008	0.008	1.6	0.000	0.045	0.045	0.0
HDL-cholesterol	−0.001	0.009	0.008	5.1	−0.002	0.046	0.044	1.1
Triglycerides	0.000	0.008	0.008	1.0	0.002	0.043	0.045	3.1
Insulin	0.000	0.008	0.008	2.4	−0.008	0.058	0.047	13.1
Outcome: hypertension								
Lifestyle factors								
Never smoked	−0.000	−0.172*	−0.172*	0.2	−0.003	−0.128*	−0.133*	2.1
Used to smoke	0.000	−0.172*	−0.172*	0.0	−0.001	−0.130*	−0.131*	0.4
Clinical measurements								
Waist-to-hip ratio	−0.006	−0.165*	−0.173*	3.4	−0.026*	−0.102*	−0.132*	19.4
Body fat percentage	−0.052*	−0.110*	−0.172*	29.8	−0.092*	−0.037	−0.131*	70.0
Cholesterol	−0.001	−0.170*	−0.171*	0.6	0.000	−0.132*	−0.132*	0.0
LDL-cholesterol	−0.001	−0.172*	−0.173*	0.6	0.000	−0.133*	−0.133*	0.0
HDL-cholesterol	0.000	−0.172*	−0.172*	−0.1	0.006	−0.139*	−0.131*	4.7
Triglycerides	−0.008*	−0.163*	−0.174*	4.7	−0.002	−0.129*	−0.132*	1.4
Insulin	0.001	−0.170*	−0.170*	0.2	0.010	−0.143*	−0.130*	7.4

The mediator is the variable hypothesised to transmit (mediate) the effect of lean diabetes on the outcome

The indirect effect estimate is the portion of the total effect of lean diabetes on the outcome that operates through the mediator

The direct effect estimate is the effect of lean diabetes on the outcome not explained by the mediator

The total effect estimate is the combined direct and indirect effects of lean diabetes on the outcome

The proportion mediated estimate is the percentage of the total effect explained by the indirect effect (i.e. mediation pathway)

Positive values indicate partial mediation; negative values may suggest inconsistent mediation or suppression effects

^a^Proportion mediated exceeds 100% because the indirect (0.028) and direct (−0.004) effects have opposite signs, indicating inconsistent mediation

^*^Indirect, direct or total effect is statistically significant at *p*≤0.05

In the RODAM study, similar patterns were observed (Table 4). Body fat percentage explained up to 70% of hypertension differences, 53% of impaired eyesight and 11% of diabetic retinopathy. Body fat percentage also accounted for about 22% of differences in 10-year CVD risk, and insulin levels contributed around 21% of CKD differences. Lifestyle factors and lipids had negligible effects (mostly <10%).

### Sensitivity analysis with geographical site

In sensitivity analyses with additional adjustment for geographical site in the RODAM cohort, findings were largely consistent with the main models (ESM Table 1, ESM Fig. 1). The associations between being lean and retinopathy, hypertension and 10-year CVD risk were unchanged, but the association with stroke was attenuated (ESM Table 1, ESM Fig. 1).

## Discussion

### Summary of key findings

Type 2 diabetes in Africans is common among individuals who are lean, yet they are typically managed similarly to those who are overweight or obese, despite potential differences in underlying biology, raising concerns about a treatment mismatch and consequent divergence in complication patterns. We aimed to determine whether complication patterns differ between these groups. Using a multi-cohort analysis of harmonised individual-level data from two large, well-characterised African studies, we found consistent differences between BMI groups. Lean participants had higher prevalence of retinopathy, impaired eyesight, and stroke, whereas those with overweight or obesity had higher hypertension and a higher estimated 10-year CVD risk. CKD did not differ between the groups. Body fat percentage accounted for a substantial proportion of these differences.

### Discussion of key findings

Type 2 diabetes in individuals who are lean affects up to 40% of Africans with type 2 diabetes [1, 3–5], compared with fewer than 10% in most high-income populations [6, 7]. It is hypothesised that type 2 diabetes in Africans who are lean may be a distinct phenotype, potentially driven predominantly by insufficient insulin secretion rather than insulin resistance [3, 4]. This raises important concerns about a potential treatment mismatch and its consequences for complication risks. To our knowledge, this is the first study to systematically assess complication and comorbidity profiles in Africans with type 2 diabetes who are lean compared with those who are overweight or obese.

Before examining complications, we confirmed that the basis for the proposed distinct type 2 diabetes phenotype in Africans who are lean (i.e. lower insulin levels and reduced beta cell function) was met [4, 19]. In both the AADM and the RODAM studies, individuals with type 2 diabetes who were lean and not using insulin had lower circulating insulin levels and markedly reduced beta cell function, consistent with previous literature suggesting that insufficient insulin secretion, rather than insulin resistance, may be the primary underlying abnormality. This formed a strong foundation for our study [4, 19]. Building on this, we observed divergent patterns of complications and comorbidities, further supporting lean type 2 diabetes as a distinct phenotype.

Retinopathy and impaired eyesight were more frequent in individuals who were lean, consistent with our hypothesis that insufficient insulin secretion may be associated with greater glucose variability, which in turn may contribute to microvascular injury [20, 21]. Both outcomes fall within the spectrum of microvascular complications [22]. Stroke was also more common in people with type 2 diabetes who were lean, although the underlying explanation remains unclear. Evidence from African stroke studies shows a high burden of haemorrhagic stroke and, among ischaemic strokes with a determined aetiology, a substantial contribution of small-vessel disease [23, 24]. However, stroke was not subtyped in our cohorts and this interpretation is hypothesis generating; future studies with detailed stroke phenotyping are needed to determine whether the excess stroke risk in individuals who are lean is driven by microvascular mechanisms. Importantly, our findings echo those from South India [19], where diabetes in individuals who are lean (defined as BMI <18.5 kg/m^2^) was associated with higher rates of microvascular complications, such as retinopathy, nephropathy and neuropathy, than in individuals who were not lean (BMI ≥18.5 kg/m^2^) [19]. Despite differences in definition, these findings collectively support the idea that a lower BMI may be linked to greater microvascular risk.

By contrast, hypertension was consistently more common and 10-year CVD risk scores were higher in people with type 2 diabetes who were overweight or obese than in those who were lean. This may reflect an atherosclerotic profile potentially associated with excess adiposity, insulin resistance and compensatory hyperinsulinaemia. The combination of visceral fat and high insulin is a well-recognised factor associated with atherosclerosis [25], providing a plausible mechanism for the higher burden of macrovascular disease in this group. On the other hand, it has also been hypothesised that people with type 2 diabetes who are lean may experience less glycaemic variability, thereby lowering their risk of macrovascular complications [21, 26]. Notably, no prior study worldwide has examined macrovascular comorbidities in lean vs non-lean type 2 diabetes, making our analysis pioneering and highlighting a potential contrast between microvascular and macrovascular disease pathways.

We did not observe clear differences in CKD prevalence between BMI groups, and the reasons for this are not entirely clear. The overall prevalence of CKD, approximately 15% in the AADM study and 10% in the RODAM study, is similar to that of other complications such as stroke, suggesting that CKD is not a rare condition. Given its commonality, we expected to detect significant differences across BMI groups. However, the lack of observed differences may be partially explained by the dual nature of kidney pathology, where both microvascular and macrovascular mechanisms contribute [22, 27]. It is possible that an excess of microvascular disease in lean type 2 diabetes is offset by an excess of macrovascular pathology in those with obesity, potentially resulting in an overall balance at the population level. On the other hand, two methodological limitations may also have obscured true differences: first, CKD was defined solely on the basis of estimated glomerular filtration rate (eGFR) derived from creatinine rather than cystatin C [28], which may be more accurate in African populations; second, albuminuria was not included, potentially limiting the detection of early microvascular kidney damage. Further studies incorporating detailed renal phenotyping are needed to disentangle these mechanisms.

Although complication prevalence and lean type 2 diabetes prevalence differed between the cohorts, the direction of findings was consistent. The lower prevalence of lean type 2 diabetes in the RODAM study (23.5% vs 37% in the AADM study) reflects the higher mean BMI observed among Ghanaians living in Europe, which is driven by migration-related changes in environment and health behaviours such as diet and sedentary behaviour [29]. The higher prevalence of diabetic retinopathy and stroke in the RODAM study may be partly explained by lower rates of prior diagnosis (71.0% vs 99.5% in the AADM study) and treatment (65.4% vs 97.1% in the AADM study), suggesting potentially longer exposure to undetected hyperglycaemia, alongside more severe beta cell failure in individuals who were lean in the RODAM study (HOMA-B: 22 in the RODAM study vs 42 in the AADM study). The consistency in direction across both cohorts nonetheless supports the robustness of the overall findings.

Mediation analyses supported these findings, with body fat percentage accounting for much of the observed differences, as it represents fat mass. While BMI reflects overall body size, it does not distinguish between fat mass and lean mass [18], meaning that individuals with the same BMI may have different body compositions, affecting their risk of complications [18]. Our results suggest that fat mass may be associated with the observed complications. Higher fat mass, especially visceral fat, in people who are overweight or obese may be associated with atherosclerosis, potentially contributing to macrovascular complications [25], thereby explaining the higher prevalence of macrovascular complications in individuals who are overweight or obese. In contrast, lower visceral fat in individuals who are lean may help prevent these macrovascular complications but may still potentially predispose them to microvascular complications due to the direct effects of glucose dysregulation [21]. Although smoking is an established risk factor for stroke and was more common among participants who were lean in the AADM study (but not in the RODAM study), our mediation analysis indicates that it does not explain the higher prevalence of complications in individuals who are lean. Because the direct effect from a mediation model is mathematically equivalent to the smoking-adjusted estimate, the finding that direct effects equalled total effects suggests that these associations are independent of smoking, whether considered as a mediator or a confounder.

These findings have significant implications for clinical care and health systems in Africa. Type 2 diabetes is common among Africans who are lean, affecting up to four in ten adults with type 2 diabetes. Current management strategies, often dominated by insulin-sensitising agents such as metformin, may be suboptimal for individuals with type 2 diabetes who are lean, where the primary issue is insufficient insulin secretion. Randomised trials are urgently needed to identify the most effective diagnostic, treatment and screening strategies for Africans with type 2 diabetes who are lean.

### Strengths and limitations

This study has several strengths. It is the first to systematically compare complication patterns between people with type 2 diabetes who are lean and those who are overweight or obese, using two of the largest and most detailed African cohorts. The inclusion of participants from three countries and migrant sites enhances the generalisability of the findings. Heterogeneity was low in pooled analyses (0.0%), further strengthening confidence in the main findings.

Several limitations must be acknowledged. The cross-sectional design precludes assessment of causality or temporal relationships between phenotype and complications. However, major complications such as CKD and CVD develop over decades. Waiting for decades of longitudinal follow-up to study these differences may delay recognition of clinically important patterns, during which individuals may continue to be managed suboptimally. In this context, our cross-sectional analysis of two large, well-characterised cohorts, with consistent findings and minimal heterogeneity (*I*^2^=0.0%) across settings and populations, provides robust and timely evidence on differences in complication patterns.

Although we adjusted for treatment status as a proxy for diabetes management, we lacked detailed data on healthcare access (e.g. medication adherence and frequency of clinical visits), so residual confounding cannot be excluded. We had data on self-reported years since diabetes diagnosis, but cautiously did not adjust for it, as this variable does not reliably reflect true disease duration in our setting. Diabetes is frequently diagnosed late in sub-Saharan Africa due to limited routine screening [2, 30], and this delay is likely more pronounced in individuals who are lean who lack the visible adiposity that prompts clinical suspicion of type 2 diabetes; adjustment would therefore risk introducing differential misclassification bias between lean and overweight/obese groups, distorting findings rather than reducing confounding. We also did not adjust for HbA_1c_ (a marker of average glycaemic level), as glycaemic level likely lies on the causal pathway between adiposity phenotype and complications [21, 31], and adjustment could obscure these associations. Future longitudinal studies that better capture true disease duration, glycaemic management and healthcare access are needed to further disentangle these relationships.

Some outcomes, such as stroke, were based on self-reported medical history and may be subject to misclassification. Nevertheless, stroke is a major clinical event that is typically well recalled, and the prevalence observed in this study is consistent with prior literature [23, 24]. Additionally, visual impairment data in the AADM cohort had a relatively high proportion of missingness (up to 39%) and were addressed using multiple imputation. While this approach might introduce imputation bias, evidence from imputation bias studies suggests that MICE can produce relatively unbiased estimates even with substantial levels of missing data [17, 32]. Importantly, findings for visual impairment were consistent with those from the RODAM cohort, which had lower levels of missingness, and followed similar patterns to diabetic retinopathy, further supporting the robustness of these results.

### Conclusion

Complication patterns in Africans with type 2 diabetes who are lean appear to diverge from those seen in individuals who are overweight or obese. In Africa, where nearly 24 million people have type 2 diabetes, and two-fifths of them are lean, these findings add to growing evidence that lean type 2 diabetes may be a distinct phenotype, underscoring the urgent need for investigation into better-targeted management for this large, potentially mistreated population.

## Supplementary Information

Below is the link to the electronic supplementary material.

ESM (PDF 190 KB)

## Data Availability

The data supporting the findings of this study are available upon request from the corresponding authors (FPC and KACM) and will remain accessible for a minimum of 10 years following publication. Due to privacy and ethical considerations, the data cannot be made publicly available. Requests for access should be directed to the corresponding authors and will be reviewed by the scientific committees of the RODAM and AADM studies. Data may be shared with qualified researchers for non-commercial research purposes, including replication, meta-analysis and secondary analyses consistent with the original study aims. Access will be granted subject to approval by the relevant institutional ethics committees, execution of a data sharing agreement and compliance with the ethical guidelines of the RODAM and AADM studies and their participating institutions. There is no restriction on the nationality or affiliation of requesting researchers provided these conditions are met.

## Abbreviations

AADM: Africa America Diabetes Mellitus
(a/p)PR: (Adjusted/pooled) Prevalence ratio
CKD: Chronic kidney disease
CVD: Cardiovascular disease
eGFR: Estimated glomerular filtration rate
MICE: Multiple imputation by chained equations
RODAM: Research on Obesity and Diabetes among African Migrants
WHO: World Health Organization
