# Supplementary material for: Divergent complication patterns of type 2 diabetes in African individuals who are lean versus overweight or obese: a multi-cohort analysis
Source: Diabetologia. 2026 Aug 23;69(11):3232–47. doi: 10.1007/s00125-026-06830-2 (PMC13627184; doi:10.1007/s00125-026-06830-2)
Supplement: Supplementary file 1 [file 125_2026_6830_MOESM1_ESM.pdf]

Divergent complication patterns of type 2 diabetes in African individuals who are lean versus overweight or obese: a multi-cohort analysis

Sabrina Esmail[1], Charles Hayfron-Benjamin[2] (ORCID: 0000-0003-0175-0340), Samuel N. Darko[3] (ORCID: 0000-0002-0750-4311), Ellis Owusu-Dabo[4] (ORCID: 0000-0003-4232-4292), Erik Beune[1] (ORCID: 0000-0002-6229-0256), Silver Bahendeka[5] (ORCID: 0000-0001-8080-7872), Ayo P. Doumatey[6], Guanjie Chen[6], Charles Agyemang[1,7] (ORCID: 0000-0002-3882-7295), Adebowale A. Adeyemo[6] (ORCID: 0000-0002-3105-3231), Charles N. Rotimi[6] (ORCID: 0000-0001-5759-053X), Karlijn A. C. Meeks[1,6,8] (ORCID: 0000-0003-3032-405X), Felix P. Chilunga[1] (ORCID: 0000-0003-4083-001X)

1. Department of Public & Occupational Health, Amsterdam Public Health Institute, Amsterdam UMC – Location AMC, University of Amsterdam, Amsterdam, the Netherlands

2. Department of Physiology, University of Ghana Medical School, University of Ghana, Accra, Ghana

3. Department of Molecular Medicine, Kwame Nkrumah University of Science and Technology, Kumasi, Ghana

4. Department of Global and International Health, Kwame Nkrumah University of Science and Technology, Kumasi, Ghana

5. Department of Medicine, Uganda Martyrs University School of Medicine, Kampala, Uganda

6. Center for Research on Genomics and Global Health, National Human Genome Research Institute, National Institutes of Health, Bethesda, MD, USA

7. Division of Endocrinology, Diabetes and Metabolism, Department of Medicine, Johns Hopkins University School of Medicine, Baltimore, MD, USA

8. Division of Endocrinology, Diabetes and Nutrition, Department of Medicine, University of Maryland School of Medicine, Baltimore, MD, USA

Karlijn A. C. Meeks and Felix P. Chilunga are joint last authors.

Corresponding authors

1. Felix P. Chilunga, f.p.chilunga@amsterdamumc.nl

2. Karlijn A. C. Meeks, karlijn.meeks@som.umaryland.edu

ESM Methods

Measurements in AADM and RODAM and their harmonisation

Brief cohort descriptions

AADM: The full description of the Africa America Diabetes Mellitus (AADM) Study and its measurements has been published elsewhere.[1−3] In brief, AADM is the longest-running genetic epidemiology study of type 2 diabetes in sub-Saharan Africa. Participants have been enrolled across four phases in several locations: Nigeria (Enugu, Ibadan, Lagos), Ghana (Accra, Kumasi) and Kenya (Eldoret). Phase 1 (1997–2001) utilised an affected sibling pair design for cases, along with the recruitment of unaffected spouse controls. Phase 2 employed a family design (2001–2006). Phase 3 (2006–2013) targeted the general population, recruiting a random sample of participants. Phase 4 (2013–2016) is a longitudinal type 2 diabetes study enrolling unrelated type 2 diabetes cases and controls to enable deep phenotyping, including multi-omics assessment in relevant tissues (e.g. whole blood, adipose tissue and skeletal muscle), and to estimate the incidence of multiple comorbidities. Adults aged 18 years and above were eligible for participation. Individuals with type 2 diabetes were consecutively enrolled at local diabetes clinics, while community-based controls were recruited through household visits and mobilisation campaigns to provide a comparison group for genetic analyses. However, only participants with type 2 diabetes are considered in the present work.

RODAM: The full description of the Research on Obesity and Diabetes among African Migrants (RODAM) Study has been published elsewhere.[4] In brief, RODAM is a multi-centre, population-based, cross-sectional cohort study designed to investigate the role of migration and environment in the development of obesity and type 2 diabetes, and their cardiovascular risk factors, among a single ethnic group, Ghanaians. The baseline cross-sectional study was conducted between 2012 and 2015, while the longitudinal follow-up (RODAM-Pros) data collection took place between 2019 and 2021. The study compared participants living in rural Ghana and urban Ghana (Accra) (representing non-migrants) with first-generation Ghanaian migrants residing in three European cities: Amsterdam (the Netherlands), Berlin (Germany) and London (UK). Eligibility targeted individuals of Ghanaian origin aged 25 to 70 years. Recruitment utilised municipal population registers and community outreach in Europe, as well as a combination of door-to-door enumeration and community announcements in Ghana. Only the baseline data are used for this study to maximise the sample size for analysis and to ensure comparability with the cross-sectional AADM datasets.

Measurements

All participants in both studies underwent standardised interviews, physical examinations, venous blood sampling and complication assessments following harmonised operating procedures. Biochemical assays, including measurements of glucose, HbA1c, lipids and creatinine, were performed at the NIH Center for Global Health genomics laboratory in the United States for AADM participants, and at the clinical chemistry laboratories at Charité–Universitätsmedizin Berlin, Germany, for RODAM participants.

Demographic measurements and harmonisation

Age was recorded as a continuous variable across both studies via questionnaire. For the pooled analysis, age was harmonised as a continuous variable, measured in years.

Sex was recorded as male or female in both cohorts via questionnaire. The resulting variable was harmonised into a categorical variable (male/female) for the pooled analysis across both cohorts.

In AADM, participants were recruited in Ghana, Nigeria and Kenya. In RODAM, Ghanaian participants were recruited in Ghana as well as in European countries, including the Netherlands, Germany and the United Kingdom. To enable harmonised data pooling, recruitment site was ultimately categorised into four regions: Ghana, Nigeria, Kenya and European sites.

Education was assessed in both studies using standardised questionnaires. The AADM study initially categorised education as: Never attended/Primary, Lower Secondary, Upper Secondary, and University/Polytechnic. The RODAM study categorised education into local levels, which included “Never been to school”, “Lower secondary”, “Upper secondary” and “Higher vocational/University”. These local

levels were systematically mapped to the harmonised categories: Never been to school, Lower secondary, Upper secondary, and Higher vocational/University.

Smoking status was determined using standardised questionnaires in both cohorts. AADM participants were categorised as never smoker, former smoker or current smoker. RODAM defined smoking status similarly as never, former or current smoker at the time of examination. Smoking status was harmonised into three categories for pooled analysis: current smoker, never smoked and former smoker.

Alcohol consumption was assessed via questionnaire in both studies. In AADM, consumption was categorised as abstainer, moderate drinker or heavy drinker. In RODAM, it was categorised simply as drinker or non-drinker, or quantified specifically by grams per day. For harmonisation, alcohol consumption was converted into a binary variable: Any (drinker) and None/Never (non-drinker).

Clinical measurements and harmonisation

Body Mass Index (BMI) was calculated from height and weight measured using stadiometers and calibrated scales in light clothing without shoes. It was calculated using the standard formula: weight (kg) / height^2 (m^2). The Waist-to-Hip Ratio (WHR) was derived from direct waist and hip circumference measurements taken with a tape measure in both studies, calculated as the ratio of waist circumference (cm) to hip circumference (cm). Body fat percentage was determined using Bioelectrical Impedance Analysis (BIA) in both studies and recorded as a percentage.

Systolic and Diastolic Blood Pressure (SBP/DBP) were recorded consistently across both studies. Measurements were taken three times on the left arm after a minimum of five minutes of rest using an oscillometric device. The average of the second and third readings was used for analysis in both cohorts.

Biochemical markers and harmonisation

Total and HDL-cholesterol were measured from fasting blood samples using enzymatic colorimetric assays in both AADM and RODAM and were initially reported in mg/dl. LDL-cholesterol was directly measured using enzymatic colorimetric assays in AADM and calculated using the Friedewald equation in RODAM for participants with triglyceride levels within the valid range. All lipid measurements were harmonised and expressed in mmol/l for pooled analyses.

Estimated glomerular filtration rate (eGFR) was calculated from fasting plasma samples in both studies using the Chronic Kidney Disease Epidemiology Collaboration (CKD-EPI) 2021 race-free equation. eGFR was recorded as a continuous variable and expressed in ml/min/1.73 m^2 in both cohorts.

Fasting plasma glucose was measured from fasting samples using standardised chemistry analysers in both AADM and RODAM and initially reported in mg/dl. For harmonised analyses, values were converted and expressed in mmol/l across cohorts.

Insulin was measured from fasting serum or plasma samples in both studies. In AADM, insulin was measured using a radioimmunoassay in μU/ml. In RODAM, insulin was measured using standardised chemical analyses (typically radioimmunoassay) and was also expressed in μU/ml. For the harmonised pooled analysis, insulin concentrations were converted to SI units (pmol/l) using the standard conversion factor of 1 μU/ml = 6.0 pmol/l.

HOMA-IR (Insulin Resistance) and HOMA-B (Beta-Cell Function) were calculated using the standard Homeostasis Model Assessment (HOMA) formula in both studies.

Type 2 diabetes and complications/comorbidities harmonisation

In both AADM and RODAM, the diagnosis of type 2 diabetes was defined by meeting any of the following criteria: a fasting plasma glucose of ≥7.0 mmol/l (126 mg/dl), previous physician diagnosis, or documented use of glucose-lowering medication. Previously undiagnosed type 2 diabetes was strictly defined as meeting the biochemical criteria without any prior diagnosis or treatment history. Indicators of type 2 diabetes management included previous diabetes diagnosis, receipt of treatment, current use of oral glucose-lowering tablets, adherence to a diabetes-tailored diet, and use of insulin injections. Information was collected via standardised

questionnaires in both cohorts and harmonised into comparable categorical variables for pooled analyses. Harmonisation was achieved by recoding study-specific responses into comparable yes/no indicators of diabetes management.

For the pooled analysis, six complications and comorbidities were included: stroke, chronic kidney disease (CKD), diabetic retinopathy, impaired eyesight, hypertension, and 10-year cardiovascular disease (CVD) risk. Stroke was assessed via self-reported medical history in both AADM and RODAM. CKD was defined as eGFR <60 ml/min/1.73 m² using the CKD-EPI formula in both studies. Diabetic retinopathy was assessed through ophthalmic examination in AADM, where status was determined by specialist ophthalmologists using fundoscopy, and by self-reported medical history in RODAM. Impaired eyesight was defined as adult-acquired visual impairment developing gradually over time, not attributable to childhood conditions or accidental injury, obtained from clinical history in both cohorts. Hypertension was defined using WHO criteria (blood pressure ≥140/90 mmHg, previous diagnosis, or use of antihypertensive medication) in both studies. The 10-year CVD risk was estimated using the Atherosclerotic Cardiovascular Disease (ASCVD) risk score, incorporating age, sex, blood pressure, cholesterol, diabetes status and smoking status in both cohorts.

References

1. Ekoru K, Doumatey A, Bentley AR, et al. Type 2 diabetes complications and comorbidity in sub-Saharan Africans. EClinicalMedicine 2019;16:30–41.

2. Rotimi CN, Chen G, Adeyemo AA, et al. A genome-wide search for type 2 diabetes susceptibility genes in West Africans: the Africa America Diabetes Mellitus (AADM) Study. Diabetes 2004;53(3):838–41.

3. Rotimi CN, Dunston GM, Berg K, et al. In search of susceptibility genes for type 2 diabetes in West Africa: the design and results of the first phase of the AADM study. Ann Epidemiol 2001;11(1):51–8.

4. Agyemang C, Beune E, Meeks K, et al. Rationale and cross-sectional study design of the Research on Obesity and type 2 Diabetes among African Migrants: the RODAM study. BMJ Open 2014;4(3):e004877.

ESM Table 1 RODAM complications models additionally adjusted for geographical site (Europe vs Africa)

Diabetes complications/comorbidities	Model 0 PR (95% CI)	Model 1 PR (95% CI)	Model 2 PR (95% CI)	Model 3 PR (95% CI)
**10-year CVD risk**				
Overweight/obese (Ref)	Ref	Ref	Ref	Ref
Lean (normal weight)	0.94 (0.74–1.20)	0.88 (0.68–1.12)	0.88 (0.69–1.13)	0.86 (0.66–1.12)
**Hypertension**				
Overweight/obese (Ref)	Ref	Ref	Ref	Ref
Lean (normal weight)	0.83 (0.64–1.06)	0.81 (0.62–1.04)	0.81 (0.62–1.05)	0.95 (0.72–1.25)
**Stroke**				
Overweight/obese (Ref)	Ref	Ref	Ref	Ref
Lean (normal weight)	2.02 (1.16–3.52)	1.45 (0.90–2.27)	1.30 (0.81–2.09)	0.80 (0.49–1.31)
**Impaired eyesight**				
Overweight/obese (Ref)	Ref	Ref	Ref	Ref
Lean (normal weight)	1.53 (0.96–2.43)	1.03 (0.78–1.36)	1.03 (0.77–1.36)	0.96 (0.71–1.29)
**Chronic kidney disease**				
Overweight/obese (Ref)	Ref	Ref	Ref	Ref
Lean (normal weight)	0.70 (0.35–1.40)	0.74 (0.33–1.51)	0.90 (0.42–1.94)	0.87 (0.39–1.94)
**Diabetic retinopathy**				
Overweight/obese (Ref)	Ref	Ref	Ref	Ref
Lean (normal weight)	1.40 (1.05–1.84)	1.46 (1.09–1.94)	1.31 (0.98–1.76)	1.40 (1.02–1.93)

Reference group: overweight/obese (BMI ≥25 kg/m²). Poisson regression models with robust standard errors were used to estimate prevalence ratios (PRs) and 95% confidence intervals (CIs). Model 0 is unadjusted; Model 1 is adjusted for age, sex and education; Model 2 is additionally adjusted for diabetes treatment; Model 3 is additionally adjusted for geographical site (Europe vs Africa).

ESM Fig. 1

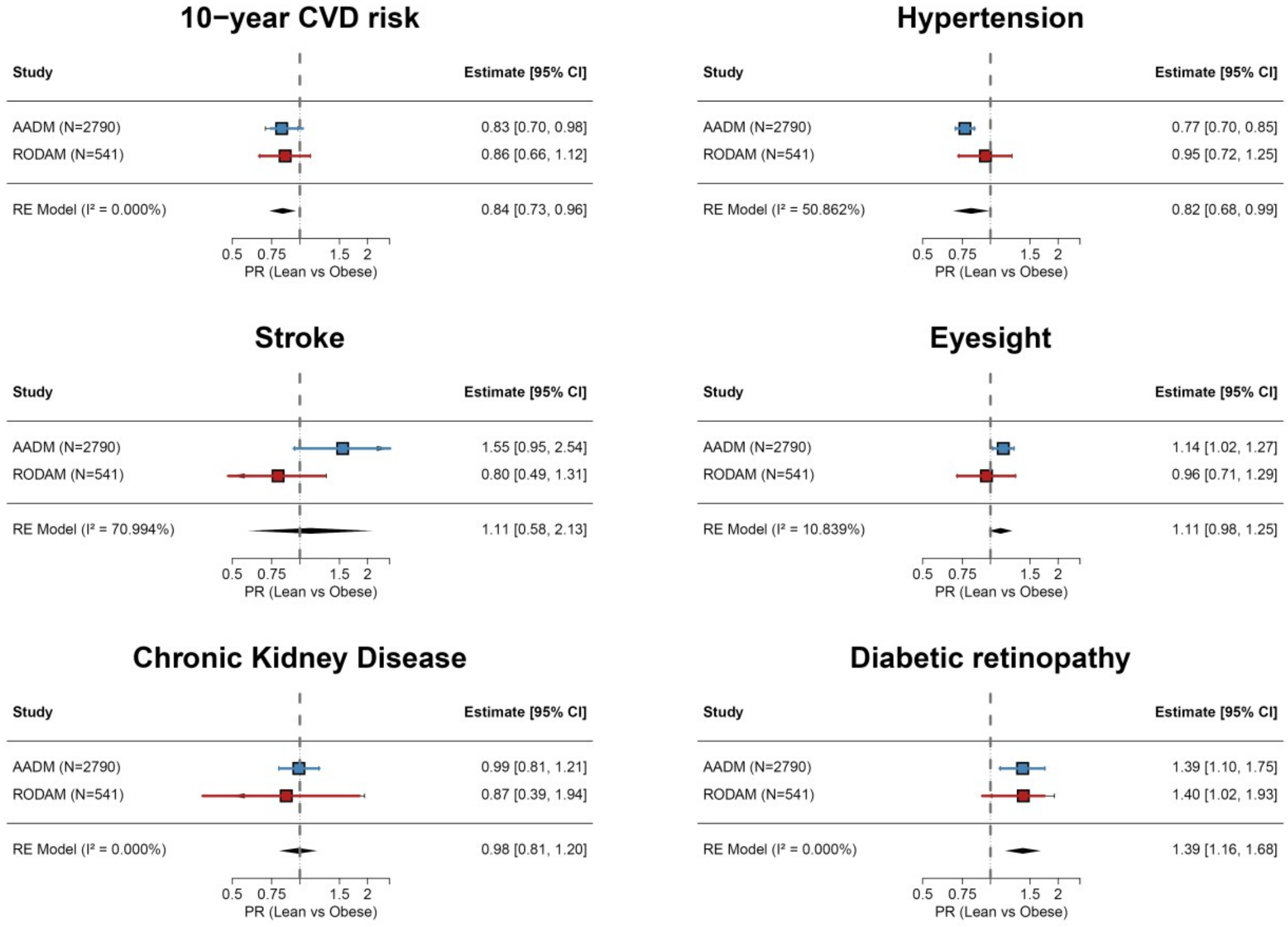

ESM Fig. 1 Pooled associations between BMI status and complications in Africans with type 2 diabetes, with additional adjustment for geographical site in RODAM. Results were largely consistent with the main analysis, though the association with stroke was attenuated following site adjustment. Prevalence ratios (PRs) with 95% confidence intervals (CIs) comparing lean individuals (BMI <25 kg/m²) with those with overweight or obesity (BMI ≥25 kg/m²), adjusted for age, sex, education, diabetes treatment and geographical site. (a) 10-year cardiovascular disease risk; (b) hypertension; (c) stroke; (d) impaired eyesight; (e) chronic kidney disease; (f) diabetic retinopathy. The dashed vertical line represents the null value (PR=1). Coloured squares represent cohort-specific estimates; the diamond represents the pooled random-effects estimate. I², heterogeneity statistic; AADM, Africa America Diabetes Mellitus study; RODAM, Research on Obesity and Diabetes among African Migrants study; PR, prevalence ratio; CI, confidence interval; BMI, body mass index.
